# Less Pronounced Immunopathological Responses Following Oral Butyrate Treatment of *Campylobacter* *jejuni*-Infected Mice

**DOI:** 10.3390/microorganisms10101953

**Published:** 2022-09-30

**Authors:** Ke Du, Minnja S. Foote, Soraya Mousavi, Agnes Buczkowski, Sebastian Schmidt, Stefan Bereswill, Markus M. Heimesaat

**Affiliations:** 1 Gastrointestinal Microbiology Research Group, Institute of Microbiology, Infectious Diseases and Immunology, Charité-Universitätsmedizin Berlin, Corporate Member of Freie Universität Berlin, Humboldt-Universität zu Berlin and Berlin Institute of Health, 12203 Berlin, Germany; 2 Hofmann & Sommer GmbH und Co., KG, Büro Berlin, 12489 Berlin, Germany

**Keywords:** butyrate, short-chain fatty acids, *Campylobacter jejuni*, immune-modulatory modes of action, host–pathogen interactions, gut microbiota depletion, campylobacteriosis model, preclinical intervention survey, antibiotic-independent natural compounds

## Abstract

Given that human *Campylobacter jejuni* infections are rising globally and antibiotic treatment is not recommended, infected patients would substantially benefit from alternative therapeutic strategies. Short-chain fatty acids such as butyrate are known for their health benefits, including anti-microbial and anti-inflammatory effects. This prompted us to investigate potential disease-alleviating properties of butyrate treatment during acute murine *C. jejuni*-induced enterocolitis. Therefore, following gut microbiota depletion IL-10^−/−^ mice were challenged with 10^9^ viable *C. jejuni* cells by oral gavage and treated with butyrate via the drinking water (22 g/L) starting on day 2 post-infection. As early as day 3 post-infection, butyrate reduced diarrheal severity and frequency in treated mice, whereas on day 6 post-infection, gastrointestinal *C. jejuni* burdens and the overall clinical outcomes were comparable in butyrate- and placebo-treated cohorts. Most importantly, butyrate treatment dampened intestinal pro-inflammatory immune responses given lower colonic numbers of apoptotic cells and neutrophils, less distinct TNF-α secretion in mesenteric lymph nodes and lower IL-6 and MCP-1 concentrations in the ileum. In conclusion, results of our preclinical intervention study provide evidence that butyrate represents a promising candidate molecule for the treatment of acute campylobacteriosis.

## 1. Introduction

*Campylobacter jejuni* are microaerophilic spiral-shaped Gram-negative bacteria and the most prevalent causes of bacterial diarrheal diseases in both developed and developing countries [1,2]. With more than 120,000 reported campylobacteriosis cases in the European Union in 2020, *C. jejuni* infections are a significant public health concern and come with high economic costs due to loss of productivity [3]. Given that *C. jejuni* is a common colonizer of farm animals, including poultry, swine, and cattle [4], most transmissions are caused by the consumption of undercooked meat and improper kitchen hygiene [5]. In addition, contaminated water or raw milk may also promote *C. jejuni* infection [5]. In humans, *C. jejuni* induces hyperactivation of the innate immune system, characterized by the invasion of the colonic mucosa and lamina propria by neutrophilic granulocytes [6]. During intestinal inflammation, *C. jejuni* causes malabsorption by disrupting tight junction proteins and impairing electrolyte homeostasis [7]. Depending on the virulence of the bacterial strain and the status of the host’s immune system, the degree of symptoms during campylobacteriosis often differs, as some individuals present relatively mild symptoms, whereas others suffer from a severe enterocolitis syndrome [2]. Main symptoms, such as watery stool with mixed blood, abdominal cramps, and fever, usually lasts 2 to 6 days. In most cases, antibiotics are not recommended, since the disease itself is self-limiting and treatment mainly focuses on rehydration with electrolyte supplementation [5,6]. However, cases with post-infectious sequelae such as Guillain–Barré syndrome or inflammatory bowel disease, for instance, have been reported within weeks or months after the primary *C. jejuni* infection [8].

The molecular basis of *C. jejuni* enteritis is directly linked to the Gram-negative bacterial cell wall molecule lipooligosaccharide (LOS), which induces a hyperactivation of the innate and adaptive immune system via Toll-like receptor-4 (TLR-4) sensing bacterial lipopolysaccharide (LPS) [9,10]. In the past, in vivo *C. jejuni* infection studies were limited by the murine microbiota, which exerts a strong colonization resistance against distinct enteropathogens, including *C. jejuni*. Moreover, conventional laboratory wild-type mice do not respond to *C. jejuni* infections since these animals are physiologically about 10,000-fold more resistant against LPS and LOS when compared with humans [11,12]. Given that interleukin-10 (IL-10) is required for LOS resistance [13], researchers use IL-10^−/−^ mice following gut microbiota depletion as a practical and reliable model for campylobacteriosis caused by *C. jejuni*-induced pro-inflammatory immune responses. After application of broad-spectrum antibiotics, mice can successfully be colonized with *C. jejuni* and exhibit, due to the lack of IL-10, symptoms of acute intestinal campylobacteriosis characterized by wasting and bloody diarrhea [14,15]. In addition, the immunopathological responses can be observed beyond the gastrointestinal tract, in extra-intestinal and systemic sites [15]. Various non-toxic molecules have already been evaluated for disease-alleviating effects during *C. jejuni* infection applying the murine infection and inflammation model, including vitamin C [16], vitamin D [17], carvacrol [18], urolithin-A [19], various essential oils [20,21,22,23], and activated charcoal [24] to date.

Infamous for its rancid smell, butyric acid (or its salt butyrate) constitutes a short chain fatty acid (SCFA) with four carbon atoms. Along with other SCFAs such as acetate and propionate, butyrate is physiologically generated by anaerobic bacterial fermentation of indigestible fiber in the intestinal lumen [25,26]. After crossing the colonic epithelia by diffusion or via monocarboxylate transporter (MCT)-1, MCT-4, and sodium-coupled monocarboxylate transporter 1 (SMCT1), butyrate can be utilized in the β-oxidation pathway. Although butyrate only constitutes the smallest percentage of all generated SCFAs in the gut, it is the primary energy source for colonocytes [25,27]. For years, it has been evident that butyrate is a crucial mediator for host-microbiome interactions and, thus, essential for maintaining intestinal homeostasis and overall gastrointestinal health [28]. Butyrate has been further shown to modulate immune functions via specific G-protein coupled receptors (GPR) such as GPR41, GPR43, GPR109A, and GPR164. Beyond the gut, these receptors can be found in various extraintestinal tissues, including fat and the nervous system, with the highest expression on immune cells [29]. For example, activation of GPR109A, highly expressed on intestinal epithelial cells, has been proven to suppress colonic inflammation, barrier damage, and even carcinogenesis [29,30,31]. Butyrate mediated anti-inflammatory properties were also linked to the downregulation of the nuclear factor kappa-light-chain-enhancer of activated B cells (NF-κB) signaling pathways and inhibition of histone deacetylase (HDAC) activity, constituting a central epigenetic control mechanism [32].

Campylobacteriosis patients may benefit substantially from novel anti-inflammatory therapies, which avoid the risk for resistance development seen in *Campylobacter* populations worldwide. Resistance to fluoroquinolones used for initially calculated therapy of bacterial infectious enteritis and for treating severe cases of *C. jejuni* infection poses a serious problem [33]. Therefore, this placebo-controlled intervention study applying a murine *C. jejuni* infection and inflammation model evaluates the multifaceted health beneficial properties of butyrate. Following gut microbiota depletion, IL-10^−/−^ mice were challenged with *C. jejuni* by oral gavage and surveyed for gastrointestinal pathogen loads, clinical outcome, and intestinal immune responses after therapeutic butyrate supplementation.

## 2. Materials and Methods

### 2.1. Ethical Statement

The murine infection study was performed under the directive 2010/63/EU (Requirements for animal protection used for scientific purposes in the European Union). All protocols were authorized by the ethical committee in Berlin (“Landesamt für Gesundheit und Soziales”, Berlin, Germany; No. G0104/19). The clinical condition of each animal was surveyed every day throughout the observation period.

### 2.2. Microbiota-Depleted IL-10^−/−^ Mice

IL-10^−/−^ mice were born and raised at the Charité University experimental mouse facility (Forschungseinrichtungen für Experimentelle Medizin, Berlin, Germany). Under specific pathogen free and standard conditions, a maximum of three mice were housed together in a cage with filter tops in a semi-barrier experimental setup (particularly a cycle of 12 h light/12 h dark, 22–24 °C, 55 ± 15% humidity). The animals were fed a standard chow diet (ssniff R/M-H, V1534-300, Sniff, Soest, Germany) and autoclaved tap water ad libitum. To assure intestinal *C. jejuni* colonization, the murine commensal intestinal microbiota had to be eradicated by an antibiotic cocktail (ABx) consisting of ampicillin plus sulbactam (2 g/L; Dr. Friedrich Eberth Arzneimittel, Ursensollen, Germany), ciprofloxacin (200 mg/L; Fresenius Kabi, Bad Homburg, Germany), imipenem (250 mg/L; Fresenius Kabi, Bad Homburg, Germany), metronidazole (1 g/L; B. Braun, Melsungen, Germany) and vancomycin (500 mg/L; Hikma Pharmaceuticals, London, UK) that was added to the autoclaved drinking water of 3-week-old mice for eight weeks, as reported previously [34,35]. To minimize cross-contamination, mice were handled under strict aseptic conditions (accessible with lab coats, sterile gloves, hair, and shoe coverings). Two days before the *C. jejuni* infection, ABx was replaced by autoclaved water to assure antibiotic washout. During the antibiotic pretreatment course as well as immediately before *C. jejuni* infection sterility of fecal samples were confirmed by culture (i.e., by using enrichment broths), as described in detail [34].

### 2.3. C. jejuni Infection

On days 0 and 1, the microbiota-depleted 3-month-old mice (total number of *n* = 36) were subjected to 10^9^ colony forming units (CFU) of *C. jejuni* strain 81–176 (oral gavage). Therefore, the enteropathogens were derived from frozen stocks and streaked onto karmali agar and on columbia agar (supplemented with 5% sheep blood) plates (both from Oxoid, Wesel, Germany) two days prior respective infections (i.e., day-2 and day-1). The culture media were incubated in a box at 37 °C (microaerophilic conditions, CampyGen gas packs, Oxoid, Wesel, Germany). Two days later, bacteria from one fluently grown karmali agar plate were harvested in 5 mL sterile phosphate-buffered saline (PBS; Thermo Fisher Scientific, Waltham, MA, USA) to an approximate McFarland density of 3 (i.e., OD of 0.6 at 600 nm wavelength) resulting in a yield of 10^9^ víable *C. jejuni* cells in a volume of 0.3 mL used for subsequent gavage. The yields were reconfirmed by cultural analyses of serial dilutions of the bacterial suspensions on respective solid culture media. The following cohorts of age- and sex-matched microbiota-depleted IL-10^−/−^ mice (individual numbers in parentheses) were finally included in three independent *C. jejuni* infection experiments: (i) naive (non-infected, non-treated) mice (6/5/5); (ii) *C. jejuni*-infected, placebo-treated mice (6/6/6); (iii) *C. jejuni*-infected, butyrate-treated mice (6/6/6).

### 2.4. Butyrate Treatment

Butyrate treatment started on day 2 post-infection (p.i.) and continued until the end of the experimental period (namely, day 6 p.i.). Sodium butyrate (Sigma-Aldrich, München, Germany) was dissolved in autoclaved tap water to a final concentration of 22 g/L and sterile filtered before being applied as a drinking solution ad libitum. Taking into consideration that the average body weight of a mouse was ∼25 g and the daily drinking volume ∼5 mL, each mouse received a daily dose of approximately 110 mg butyrate (4.4 g/kg body weight/day). The placebo group consisting of sex-matched litter mate mice was offered sterilized (i.e., autoclaved) drinking water instead (ad libitum).

### 2.5. Gastrointestinal Colonization of C. jejuni

After the oral challenge, bacterial loads were quantitatively surveyed in feces samples daily, and upon sacrifice on day 6 p.i., gastrointestinal luminal contents were derived under aseptic conditions. Therefore, fecal contents from the colon, ileum, duodenum, and stomach lumen were carefully squeezed into 2.0 mL plastic tubes containing 1.0 mL sterile PBS (Thermo Fisher Scientific, Waltham, MA, USA). The weights of respective tubes were assessed before and after careful transfer of the fecal luminal samples. Then, serial dilutions of fecal aliquots were plated on columbia agar plates (Oxoid, Wesel, Germany), and incubated for 48 h at 37 °C under microaerophilic conditions. The CFU numbers were normalized to the weight of the fecal sample as assessed by the difference in weights of the plastic tubes after and before transfer of respective sample. The detection limit of viable *C. jejuni* was 100 CFU/g.

### 2.6. Clinical Outcome

To quantify the clinical outcome of *C. jejuni*-induced disease, we assessed different parameters during experimental period each day. The cumulative clinical scores (maximum 12 points) are composed of fecal blood (0: no blood; 2: microscopic detection of blood applying the Guaiac-based fecal occult blood testing (Haemoccult, Beckman Coulter/PCD, Krefeld, Germany); 4: macroscopic blood visible), diarrhea (0: normal feces; 2: pasty feces; 4: liquid feces) and clinical symptoms including wasting aspects (0: normal; 2: ruffled fur, reduced locomotion; 4: isolation, severely impaired locomotion, pre-final aspect).

### 2.7. Sampling Methods

Six days after *C. jejuni* infection, mice were sacrificed by carbon dioxide asphyxiation. Ex vivo biopsies of mesenteric lymph nodes (MLN), colon, and ileum were taken under aseptic conditions for cytokine measurements. In parallel, gastrointestinal samples from the colon, ileum, duodenum, and stomach were preserved for subsequent cultural analyses. Furthermore, colonic samples were derived for quantitative in situ immunohistochemical detection of distinct immune cell populations.

### 2.8. Immunohistochemistry

Immunohistochemical stainings were performed in ex vivo colonic samples that had been fixed in 5% formalin and embedded in paraffin, as previously reported [36]. In brief, to detect apoptotic cells, neutrophils and regulatory T cells, as well as T and B lymphocytes, the paraffin sections were stained with primary antibodies against cleaved caspase-3 (Asp175, Cell Signaling, Beverly, MA, USA, 1:200), MPO7 (No. A0398, Dako, Glostrup, Denmark, 1:500), FOXP3 (clone FJK-165, No. 14-5773, eBioscience, San Diego, CA, USA, 1:100), CD3 (No. N1580, Dako, Glostrup, Denmark, 1:10), and B220 (No. 14-0452-81, eBioscience, San Diego, CA, USA, 1:200), respectively. An independent investigator determined the mean numbers of positive cells in 6 high-power fields (HPF, 0.287 mm^2^; 400× magnification).

### 2.9. Pro-Inflammatory Mediator Measurements

Longitudinally cut and washed (in PBS) colonic and ileal ex vivo biopsies (strips of approximately 1 cm^2^), as well as ex vivo biopsies derived from the MLN (3 nodes), were cultured for 18 h at 37 °C in 24-flat-bottom well culture plates (Thermo Fisher Scientific, Waltham, MA, USA) containing 500 μL serum-free RPMI 1640 medium (Thermo Fisher Scientific, Waltham, MA, USA), plus penicillin (100 U/mL; Biochrom, Berlin, Germany) and streptomycin (100 μg/mL; Biochrom, Berlin, Germany). Applying the Mouse Inflammation Cytometric Bead Array (CBA; BD Biosciences, Heidelberg, Germany) on a BD FACSCanto II flow cytometer (BD Biosciences, Heidelberg, Germany), respective culture supernatant samples were tested for interleukin-6 (IL-6), tumor necrosis factor-alpha (TNF-α), and monocyte chemoattractant protein-1 (MCP-1) according to the manufacturer’s instructions.

### 2.10. Data Analysis

Data were pooled from three independent experiments. Prism (version 9, GraphPad, San Diego, CA, USA) was used to assess medians and significance levels (two-sided probability (*p*) values ≤ 0.05) by applying Student’s *t*-test and the Mann–Whitney test for pairwise comparisons of normally and not normally distributed data, respectively. For multiple comparisons, the one-sided ANOVA with Tukey’s post-correction (for normally distributed data) and the Kruskal–Wallis test with Dunn’s post-correction (for not normally distributed data) were applied.

## 3. Results

### 3.1. Intestinal Pathogen Loads following Oral Butyrate Treatment of C. jejuni-Infected Mice with Acute Enterocolitis

**Figure 1:**
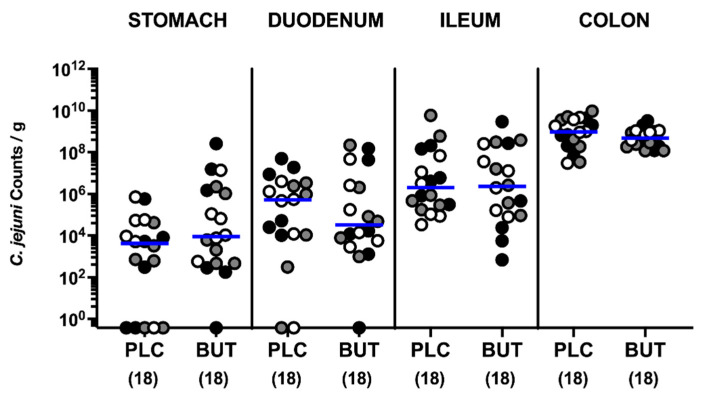
Gastrointestinal *C. jejuni* colonization in infected IL-10^−/−^ mice upon butyrate treatment. Following gut microbiota depletion IL-10^−/−^ mice were challenged with *C. jejuni* strain 81–176 by oral gavage on days 0 and 1. Starting from day 2 p.i., the animals were subjected to butyrate (BUT) or placebo (PLC) via the autoclaved tap water ad libitum. On day 6 p.i. the pathogen numbers were assessed in luminal samples derived from the stomach, duodenum, ileum, and colon by culture and indicated as *C. jejuni* counts per gram. Differently colored circles indicate data from three individual experiments. Medians (blue bar) and the total numbers of included animals (parentheses) are shown.

We first addressed whether oral butyrate treatment would impact *C. jejuni* colonization within the gastrointestinal tract of infected mice. On two consecutive days (namely, days 0 and 1), microbiota-depleted IL-10^−/−^ mice were perorally challenged with 10^9^ viable *C. jejuni* cells by gavage. Starting from day 2 p.i. and lasting until necropsy, the animals were subjected to butyrate via the drinking water (ad libitum). Daily cultural analyses of *C. jejuni* in fecal samples revealed that butyrate did not lower fecal pathogen burdens (Figure S1). On day 6 p.i., *C. jejuni* numbers derived from defined parts of the gastrointestinal lumen were comparable between butyrate- and placebo-treated mice (Figure 1). Hence, oral butyrate treatment of *C. jejuni*-infected mice does not affect gastrointestinal pathogenic colonization.

### 3.2. Clinical Outcome following Oral Butyrate Treatment of C. jejuni-Infected Mice with Acute Enterocolitis

**Figure 2:**
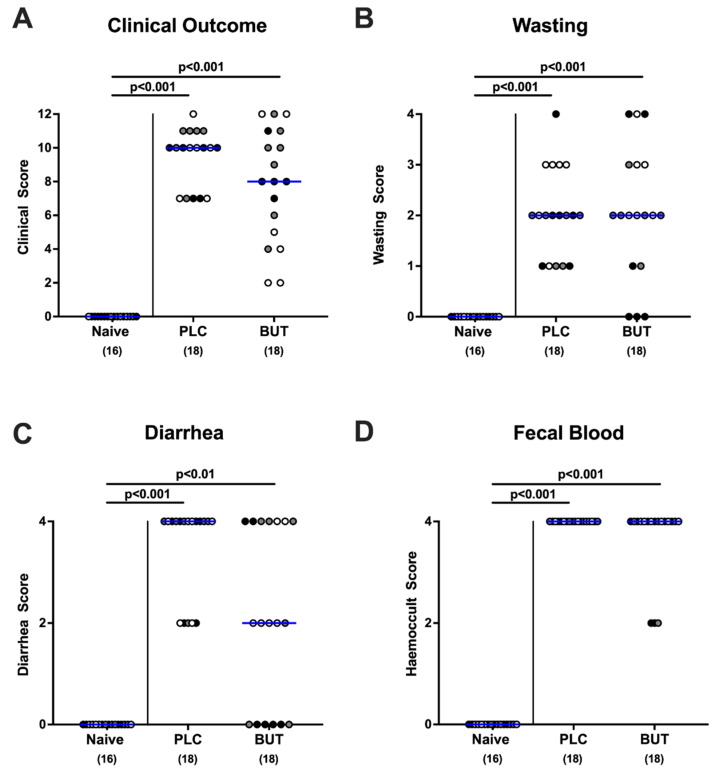
Clinical outcome of *C. jejuni*-infected mice upon butyrate treatment. Following gut microbiota depletion, IL-10^−/−^ mice were challenged with *C. jejuni* strain 81–176 by oral gavage on days 0 and 1. Starting from day 2 p.i., the animals were subjected to butyrate (BUT) or placebo (PLC) via the autoclaved tap water ad libitum. Naive mice served as uninfected and untreated controls. (**A**) The overall clinical outcome was quantitated in all animals on day 6 p.i. with a clinical scoring system by adding scores of distinct parameters including (**B**) wasting, (**C**) diarrhea, and (**D**) fecal blood. Differently colored circles indicate data from three individual experiments. Medians (blue bar), significance levels (*p* values) evaluated by the ANOVA test with Tukey post-correction or by the Kruskal–Wallis test and Dunn’s post-correction, and total numbers of included animals (parentheses) are shown.

**Figure 3:**
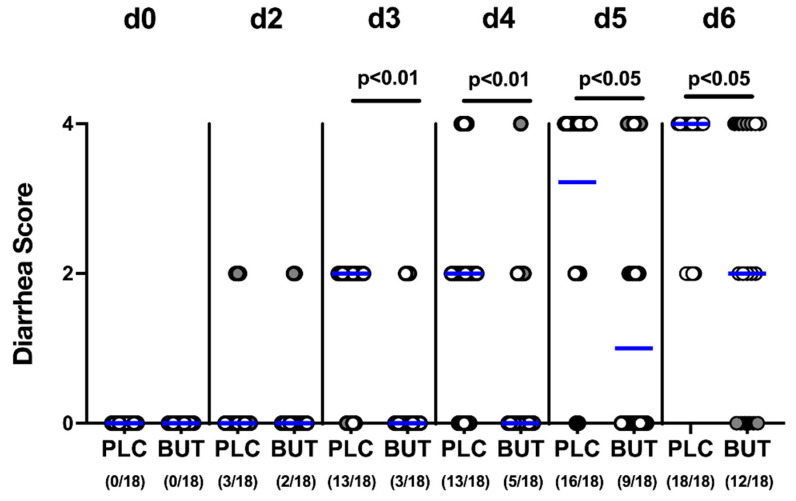
Diarrhea development over time in *C. jejuni*-infected mice upon butyrate treatment. Following gut microbiota depletion IL-10^−/−^ mice were challenged with *C. jejuni* strain 81–176 by oral gavage on days 0 and 1. Starting from day 2 p.i., the animals were treated with either butyrate (BUT) or placebo (PLC) via the autoclaved tap water ad libitum. Diarrheal severity was assessed in each animal from day 0 to day 6 p.i. applying a clinical diarrhea score. Differently colored circles indicate data from three individual experiments. Medians (blue bar), significance levels (*p* values) evaluated by the Mann–Whitney test, and the number of diarrheal mice out of the total cohort in parentheses are given.

Upon sacrifice (day 6 p.i.), mice from both cohorts exhibited a similar outcome of acute campylobacteriosis as indicated by comparable clinical scores (n.s.; Figure 2). As early as day 3 p.i. and 24 h after initiation of treatment, butyrate-treated mice suffered less distinctly from diarrhea compared with the placebo group, given lower clinical diarrhea scores in the former versus the latter (*p* < 0.01–0.05; Figure 3). Notably, one-third of the butyrate-treated mice did not display any diarrheal symptoms, whereas all placebo counterparts were suffering from diarrhea. Thus, butyrate treatment alleviates the development of diarrhea during acute campylobacteriosis.

### 3.3. Effects of Oral Butyrate Treatment on C. jejuni-Induced Apoptosis in Mice with Acute Enterocolitis

**Figure 4:**
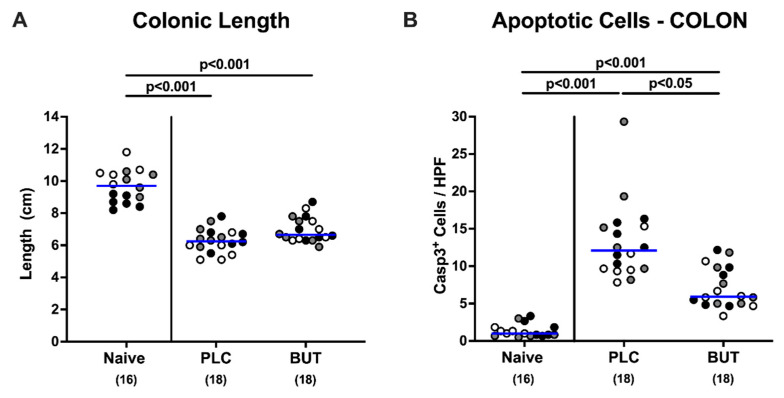
Colonic lengths and apoptotic cells in colonic epithelia upon oral butyrate treatment. Following gut microbiota depletion, IL-10^−/−^ mice were challenged with *C. jejuni* strain 81–176 by oral gavage on days 0 and 1. Starting from day 2 p.i., the animals were subjected to butyrate (BUT) or placebo (PLC) via the autoclaved tap water ad libitum. Naive mice served as uninfected and untreated controls. After the sacrifice of the mice on day 6 p.i., (**A**) colonic lengths were measured and (**B**) apoptotic epithelial cells were counted in each colonic paraffin section after immunohistochemical staining with anti-cleaved caspase3 (Casp3^+^). The mean of six counts out of representative high-power fields (HPF, 400-fold magnification) per individual mouse was applied. Differently colored circles indicate data from three individual experiments. Medians (blue bar), significance levels (*p* values) evaluated by the ANOVA test with Tukey post-correction or by the Kruskal–Wallis test and Dunn’s post-correction, and total numbers of included animals (parentheses) are shown.

Since intestinal inflammation during *C. jejuni* mediated acute enterocolitis is associated with significantly shorter intestines, we measured the colonic lengths after sacrificing the mice. Murine campylobacteriosis was accompanied by shorter colons in infected compared with uninfected mice (*p* < 0.001; Figure 4A), whereas the large intestinal lengths did not differ between butyrate and placebo cohorts (n.s.; Figure 4A). We further quantitated the numbers of cleaved caspase3^+^ colonic epithelial cells, indicative of apoptosis and a microscopic marker for intestinal inflammatory responses during murine campylobacteriosis. *C. jejuni* infection resulted in markedly increased numbers of apoptotic colonic epithelial cells (*p* < 0.001 versus naive; Figure 4B and Figure S2A). This increase was, however, far less pronounced upon butyrate treatment as indicated by more than 50% lower caspase3^+^ colonic epithelial cells in mice from the butyrate versus the placebo cohort on day 6 p.i. (*p* < 0.05; Figure 4B and Figure S2A). Hence, butyrate treatment dampens *C. jejuni*-induced apoptosis in colon epithelial cells.

### 3.4. Immunomodulatory Effects of Oral Butyrate Treatment in C. jejuni-Infected Mice with Acute Enterocolitis

**Figure 5:**
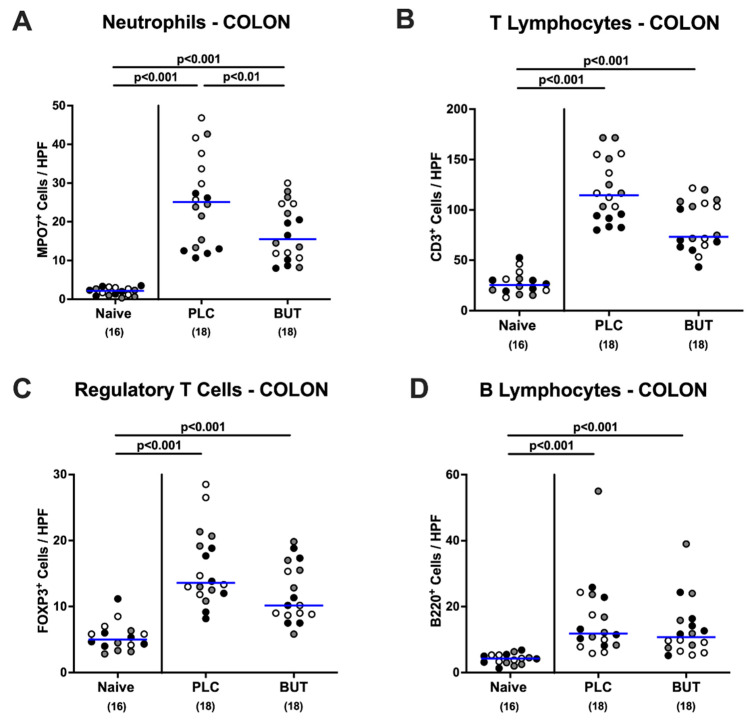
Immune cell populations in the colon upon butyrate treatment of *C. jejuni* mice. Following gut microbiota depletion IL-10^−/−^ mice were challenged with *C. jejuni* strain 81–176 by oral gavage on days 0 and 1. Starting from day 2 p.i., the animals were subjected to butyrate (BUT) or placebo (PLC) via the autoclaved tap water ad libitum. Naive mice served as uninfected and untreated controls. On day 6 p.i., (**A**) neutrophils, (**B**) T lymphocytes, (**C**) regulatory T cells, and (**D**) B lymphocytes were counted in the mucosa and lamina propria of immunohistochemically stained large intestinal paraffin sections (median counts from six high-power fields (HPF), 400-fold magnification). Differently colored circles indicate data from three individual experiments. Medians (blue bar), significance levels (*p* values) evaluated by the ANOVA test with Tukey post-correction or by the Kruskal–Wallis test and Dunn’s post-correction, and the total numbers of included animals (parentheses) are shown.

We quantitively assessed innate and adaptive immune cell responses in the large intestinal tract following oral butyrate application to *C. jejuni*-infected mice and performed immunohistochemical stainings of colonic paraffin sections. *C. jejuni* infection resulted in increased numbers of MPO7^+^ cells in the colonic mucosa and lamina propria, indicative of neutrophilic granulocytes as members of the innate immune system (*p* < 0.001 versus naive; Figure 5A and Figure S2B). Butyrate-treated mice, however, exhibited approximately 40% lower numbers of colonic neutrophils on day 6 p.i. when compared to the placebo control group (*p* < 0.01; Figure 5A and Figure S2B). This was also the case for CD3^+^ T lymphocytes counted in the colonic mucosa and lamina propria, but the difference did not reach statistical significance due to respective standard deviations (n.s.; Figure 5B and Figure S2C). In addition, mice from both cohorts displayed comparably elevated colonic numbers of FOXP3^+^ regulatory T cells and B220^+^ B lymphocytes on day 6 p.i. (*p* < 0.001 versus naive; Figure 5C,D and Figure S2D,E). Hence, oral butyrate treatment resulted in less *C. jejuni*-induced accumulation of neutrophils in the large intestinal tract of infected mice.

### 3.5. Intestinal Pro-Inflammatory Mediator Secretion following Oral Butyrate Treatment of C. jejuni-Infected Mice with Acute Enterocolitis

**Figure 6:**
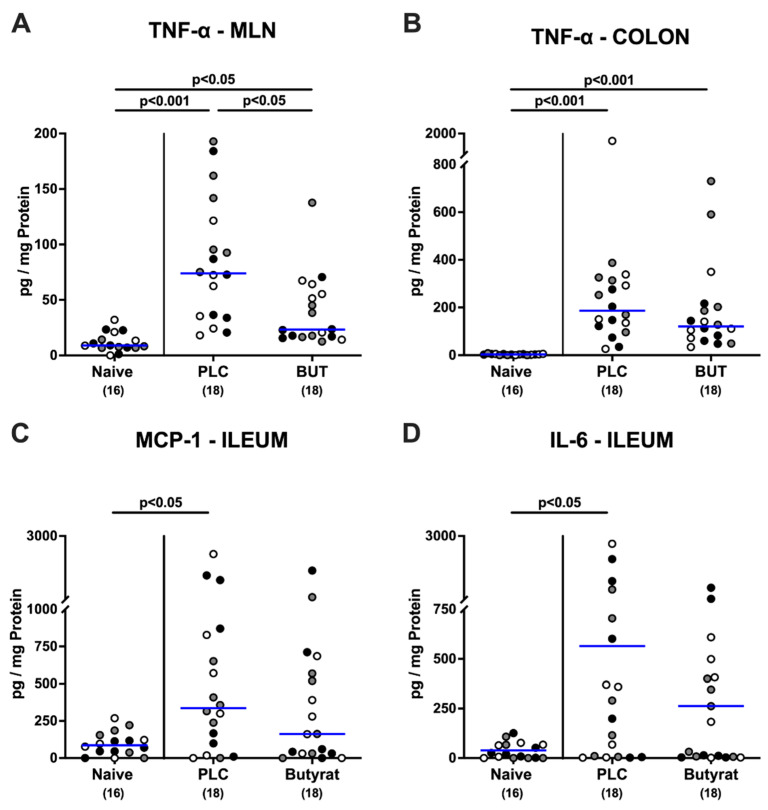
Intestinal pro-inflammatory mediator secretion upon therapeutic butyrate application to *C. jejuni*-infected mice. Following gut microbiota depletion IL-10^−/−^ mice were challenged with *C. jejuni* strain 81–176 by oral gavage on days 0 and 1. Starting from day 2 p.i., the animals were subjected to butyrate (BUT) or placebo (PLC) via the autoclaved tap water ad libitum. Naive mice were uninfected and untreated, and served as control animals. On day 6 p.i., TNF-α secretion was assessed in ex vivo biopsies derived from (**A**) mesenteric lymph nodes (MLN) and (**B**) colon, whereas in the terminal ileum, (**C**) MCP-1 and (**D**) IL-6 concentrations were measured. Differently colored circles indicate data from three individual experiments. Medians (blue bar), significance levels (*p* values) evaluated by the ANOVA test with Tukey post-correction or by the Kruskal–Wallis test and Dunn’s post-correction, and total numbers of included animals (parentheses) are shown.

We then investigated pro-inflammatory mediator responses in distinct parts of the intestinal tract upon butyrate treatment of *C. jejuni*-infected mice. Six days after oral challenge with *C. jejuni*, elevated TNF-α concentrations were measured in ex vivo biopsies taken from the MLN and colon (*p* < 0.05–0.001 versus naive; Figure 6A,B). TNF-α secretion in the MLN of butyrate-treated mice was significantly less pronounced when compared to their placebo counterparts on day 6 p.i. (*p* < 0.05; Figure 6A), which, however, did not hold true for the colon (n.s.; Figure 6B). In addition, elevated IL-6 and MCP-1 concentrations could be assessed in the ileum of placebo (*p* < 0.01 and *p* < 0.05, respectively) but not in butyrate-treated mice when compared to naive control animals (Figure 6C,D). Hence, therapeutic application of butyrate to mice with acute campylobacteriosis results in less pronounced intestinal secretion of pro-inflammatory mediators.

## 4. Discussion

In our clinical intervention study applying an acute campylobacteriosis in vivo model, therapeutic butyrate treatment via the oral route resulted in reduced intestinal inflammatory sequelae, whereas gastrointestinal *C. jejuni* loads were not affected in infected microbiota-depleted IL-10^−/−^ mice. The butyrate concentration within the drinking solution (i.e., 22,000 mg/L) was more than five times above the minimal inhibitory concentration (MIC) of 4096 mg/L determined for the *C. jejuni* strain 81–176 (data not shown). The fact that butyrate treatment did not exert clinically relevant antimicrobial activity against *C. jejuni* is most possibly due to the short half-life of butyrate in vivo [37]. However, several in vitro studies have proven the antibacterial effects of butyrate against *C. jejuni* and other Gram-negative bacteria such as *Salmonella* [38,39]. In addition, given the mixing and dilutional effects of intestinal fluids, the concentration of the biologically active molecule within the infected intestines can be expected to be much lower than the MIC measured in vitro.

Furthermore, one needs to take into consideration that the duration of the butyrate treatment was relatively short. Thus, prophylactic butyrate treatment regimens might exert more pronounced antibacterial effects in vivo and therefore need to be addressed in future studies. It is further tempting to speculate that most of the molecule has been reabsorbed in the upper gastrointestinal tract, whereas the concentration of biologically active butyrate reaching the colonic lumen was far too low to exhibit relevant antibacterial effects. Thus, augmentation of intestinal butyrate production by probiotic supplementation or application of the SCFA in a formulation facilitating release in the colon might be more effective in achieving pathogen-lowering effects in the distal intestines [40,41].

Despite the high *C. jejuni* loads in the colon, butyrate-treated mice suffered less distinctly from diarrhea during acute campylobacteriosis. Importantly, butyrate application prevented diarrhea in one-third of the mice, not showing any changes in stool consistency starting as early as day 3 p.i. and lasting until termination of the study. Previous studies have shown that butyrate regulates the expression of tight junction proteins such as claudin-3, occluding, and ZO-1, as well as intestinal mucus production [42,43,44]. Therefore, we hypothesize that *C. jejuni*-induced disruption of epithelial tight junction proteins might have been alleviated through butyrate by upregulation of tight junction protein expression preserving the colonic epithelial barrier [7,44]. The transfer of the results obtained in our preclinical placebo-controlled intervention study to defined treatment options during human campylobacteriosis is supported by the fact that the anti-diarrheal effects of butyrate have been confirmed in humans recently. In line with a past clinical trial, sodium butyrate supplementation reduced the occurrence of travelers’ diarrhea and prevented acute dehydration following diarrheal diseases [45]. A recent in vitro study addressing butyrate pretreatment of Caco-2 cells during *C. jejuni* infection further showed that butyrate prevented pathogenic adhesion and preserved butyrate receptors and transporters, namely GPR109A and SMCT1, necessary for water and electrolyte absorption and anti-inflammatory signaling pathways [46].

The diarrhea-alleviating effects in *C. jejuni*-infected IL-10^−/−^ mice upon oral butyrate treatment were accompanied by lower numbers of apoptotic colonic epithelial cells. In support, butyrate even protected rats from neuronal apoptosis during middle cerebral artery occlusion via GPR41 activation [47]. Moreover, butyrate-treated mice exhibited less severe immune cell responses as indicated by significantly lower numbers of neutrophils in the colonic mucosa and lamina propria during campylobacteriosis. In contrast, at least a trend towards reduced adaptive immune cell populations such as T and B lymphocytes could be observed when compared to infected placebo counterparts. This is in line with a previous in vivo study showing less distinct recruitment of neutrophils and reduced inflammation after the application of a butyrate-releasing derivative [48,49]. We could further show that oral butyrate treatment resulted in less pronounced TNF-α secretion in MLN draining the infected and inflamed intestines. Our results are well in line with previous studies, showing that butyrate decreases bacteria-induced inflammation by reducing pro-inflammatory cytokines such as TNF-α upon inhibition of neutrophilic infiltration [48,50]. In addition, elevated MCP-1 and IL-6 concentrations were measured in the terminal ileum of placebo, but not of butyrate-treated mice at day 6 p.i. Although the molecular mechanisms underlying the disease-alleviating properties of butyrate observed in our present preclinical intervention study have not been investigated in more detail, we would like to propose that the anti-inflammatory effects might have been due to down-regulating events within NFκB-dependent signaling pathways [42]. Considering that butyrate exhibits anti-inflammatory properties and can also preserve intestinal epithelial barrier functions, dampening the excessive immune responses following *C. jejuni* infection constitutes a promising novel therapeutic approach to combat acute campylobacteriosis [31,51]. Furthermore, since past studies have linked decreases in SCFAs to be critical in the pathogenesis of inflammatory bowel diseases [52], constituting post-infectious sequelae of *C. jejuni* infection, it is tempting to speculate that butyrate treatment might also alleviate the occurrence of post-infectious complications due to its anti-apoptotic and putative intestinal barrier preserving properties. Another area of future research to elevate butyrate mediated effects is to combine the SCFAs with other antibacterial or immunomodulatory compounds. In vitro studies have suggested that butyrate exhibits synergistic anti-microbial and anti-inflammatory effects when applied with different organic or plant-derived compounds such as forskolin, for instance [53,54]. Hence, the disease-alleviating effects of butyrate during *C. jejuni* infection might be further enhanced synergistically when combined with the yet-to-be-identified compound(s) in effective dosages.

The preclinical results in the here applied murine *C. jejuni* infection and inflammation model demonstrate that butyrate may represent a novel option for enteritis treatment in patients suffering from campylobacteriosis. The anti-inflammatory effects of butyrate may also lower the risk for post-infectious sequelae of acute campylobacteriosis. The application of butyrate is considered safe given a low risk of potential adverse effects and circumvents the risk of resistance development associated with antibiotic treatment. Since butyrate displays a strong smell, compliance of patients can be assured by applying odorless butyrate analogs in capsules [55]. In addition, patients may benefit from high fiber diets to boost bacterial butyrate production in the gut [56].

## 5. Conclusions

Our placebo-controlled intervention study provides evidence that butyrate is a promising candidate molecule to ameliorate enteric inflammation during acute campylobacteriosis.

## Data Availability

The corresponding author provides data upon request.

## Supplementary Materials

The following supporting information can be downloaded at: https://www.mdpi.com/article/10.3390/microorganisms10101953/s1, Figure S1: Kinetic survey of pathogen loads in *C. jejuni*-infected IL-10^−/−^ mice following butyrate treatment. Figure S2: Representative photomicrographs illustrating quantitative in situ immunohistochemical analyses of (A) apoptotic epithelial cells (positive for cleaved caspase-3) and distinct (B) innate (MPO7^+^ neutrophilic granulocytes) as well as adaptive immune cell subsets such as (C) CD3+ T lymphocytes, (D) FOXP3+ regulatory T cells, and (E) B220+ B lymphocytes in stained colonic paraffin sections.
