# Supplementary material for: Less Pronounced Immunopathological Responses Following Oral Butyrate Treatment of Campylobacter jejuni-Infected Mice
Source: Microorganisms. 2022 Sep 30;10(10):1953. doi: 10.3390/microorganisms10101953 (PMC9609162; doi:10.3390/microorganisms10101953)
Supplement: Supplementary file 1 [file microorganisms-10-01953-s001.zip › microorganisms-1843268-supplementary.pdf]

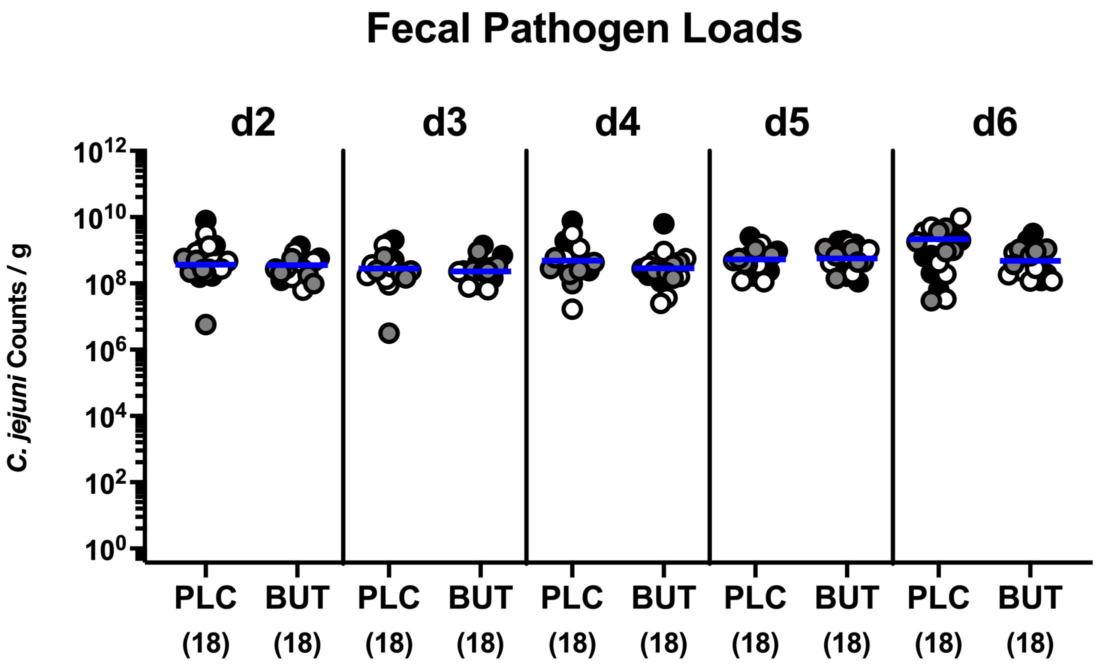

Supplementary Figure S1. Kinetic survey of pathogen loads in *C. jejuni* infected IL-10$^{-/-}$ mice following butyrate treatment. Microbiota-depleted IL-10$^{-/-}$ mice were perorally infected with *C. jejuni* strain 81-176 on days 0 and 1. Starting from day 2 p.i. the animals were subjected to butyrate (BUT) or placebo (PLC) via the drinking water *ad libitum*. On day 6 p.i., the pathogen numbers were determined in different luminal samples derived from the stomach, duodenum, ileum, and colon by culture and indicated as *C. jejuni* counts per gram. Differently colored circles indicate data from three individual experiments. Medians (blue bar) and the total numbers of included mice (in parentheses) are shown.

Supplementary Figure S2. Representative photomicrographs illustrating quantitative *in situ* immunohistochemical analyses of **(A)** apoptotic epithelial cells (positive for cleaved caspase-3) and distinct **(B)** innate (MPO7+ neutrophilic granulocytes) as well as adaptive immune cell subsets such as **(C)** CD3+ T lymphocytes, **(D)** FOXP3+ regulatory T cells, and **(E)** B220+ B lymphocytes in stained colonic paraffin sections.

A

Apoptotic Cells (COLON)

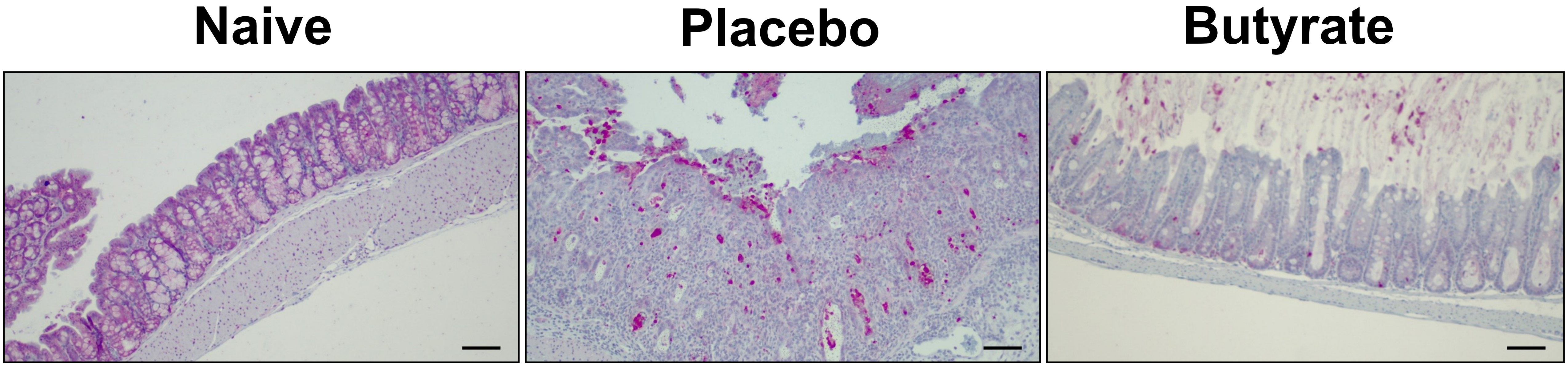

(100 x magnification, scale bar 100 µm)

B

Neutrophils (COLON)

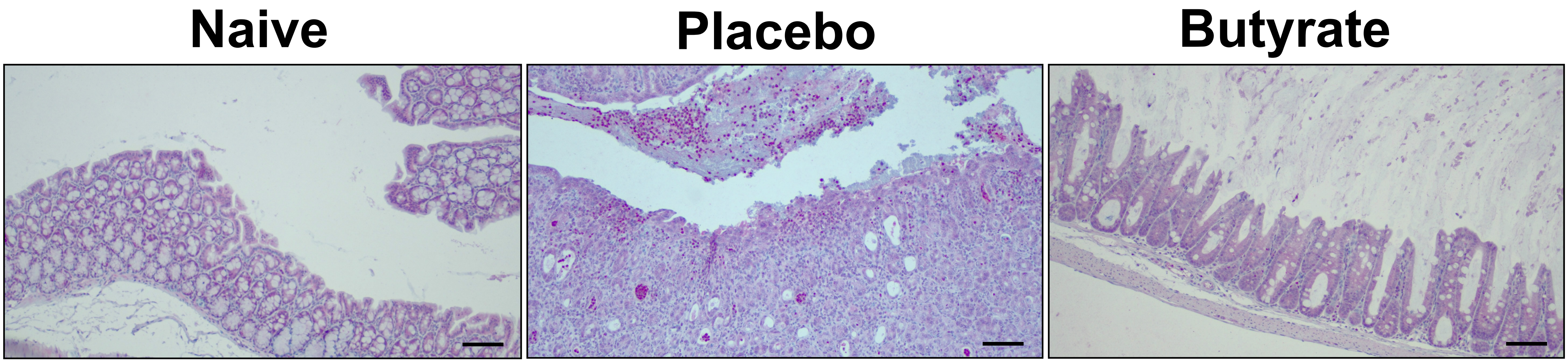

(100 x magnification, scale bar 100 μm)

C

T Lymphocytes (COLON)

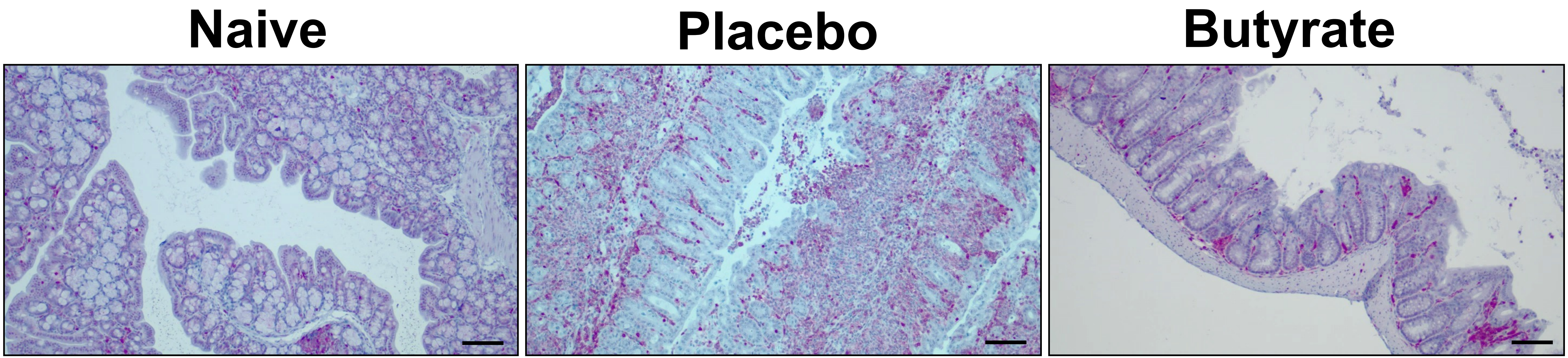

(100 x magnification, scale bar 100 µm)

D Regulatory T Cells (COLON)

Naive Placebo Butyrate

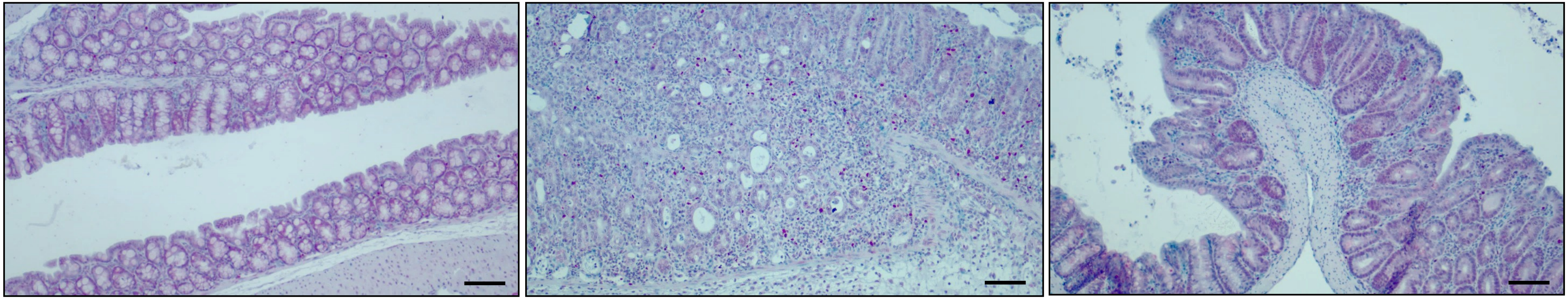

(100 x magnification, scale bar 100 µm)

E

B Lymphocytes (COLON)

Naive | Placebo | Butyrate

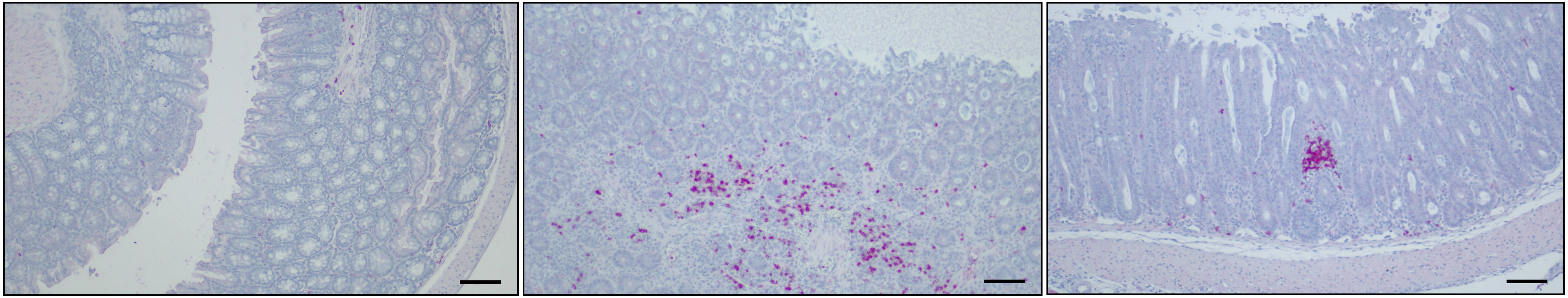

(100 x magnification, scale bar 100 µm)
